# Effect of CuO on the Structural, Antimicrobial, and Redox Activity of TiO_2_/TeO_2_/CuO Sol–Gel Powders

**DOI:** 10.3390/gels12030253

**Published:** 2026-03-18

**Authors:** Kalina Ivanova, Elitsa Pavlova, Iva Kirova, Iliana A. Ivanova, Albena Bachvarova-Nedelcheva

**Affiliations:** 1 Institute of General and Inorganic Chemistry, Bulgarian Academy of Sciences, Acad. G. Bonchev Str., Bl. 11, 1113 Sofia, Bulgaria; albenadb@svr.igic.bas.bg; 2 National Centre of Excellence Mechatronics and Clean Technologies, 8 Bul., Kl. Ohridski, 1756 Sofia, Bulgaria; 3 Faculty of Physics, Sofia University “St. Kliment Ohridski”, 5 James Bourchier Blvd., 1164 Sofia, Bulgaria; elli_pavlova@abv.bg (E.P.); iva_kirova88@abv.bg (I.K.); 4 Center of Competence “Clean Technologies for Sustainable Environment—Water, Waste, Energy for Circular Economy”, 1000 Sofia, Bulgaria; 5 Faculty of Biology, Sofia University “St. Kliment Ohridski”, 8 Dragan Tsankov Blvd., 1164 Sofia, Bulgaria; iaivanova@biofac.uni-sofia.bg

**Keywords:** sol–gel, thermal treatment, antimicrobial activity, ROS, chemiluminescence

## Abstract

This study investigates the synthesis, characterization, antimicrobial performance, and redox activity of sol–gel–derived TiO_2_/TeO_2_/CuO powders. The as-prepared gel with the nominal composition 80TiO_2_/10TeO_2_/10CuO was subjected to thermal treatment at 400 °C and 600 °C for 2 h, resulting in the formation of composite materials at both temperatures. By UV-Vis spectroscopy, it has been found that CuO is responsible for the red shifting of the absorption edge. The SEM-EDS analysis verified the elemental composition of the synthesized powders. The antimicrobial activity of the heat-treated powders was proved against *Escherichia coli* ATCC 25922 and *Staphylococcus aureus* ATCC 25923, representative Gram-negative and Gram-positive bacteria frequently associated with hospital-acquired infections and antibiotic resistance. At physiological pH, the 600 °C-treated sample exhibited strong prooxidant properties, supporting antimicrobial activity. At alkaline conditions, the nanomaterials were effective against superoxide radicals. The variation in oxidation with changes in pH is indicative of the potential for controlled application. Antimicrobial activity was assessed through minimum inhibitory concentration (MIC) assays and spot and luminescent tests, providing both quantitative and qualitative evaluations.

## 1. Introduction

Metal oxide materials have long been known for their ability to interact with microbial systems by producing reactive oxygen species (ROS) through redox reactions and surface chemistry [1]. These inorganic materials provide superior thermal and chemical stability, resistance to degradation, and the flexibility to customize functional qualities through structural and compositional control [2,3,4]. Consequently, nanostructured metal oxides have garnered increasing attention for their antimicrobial applications in protective surface coatings, environmental remediation, and biomedical devices [5,6].

By incorporating oxides with different functional roles, hybrid metal oxide systems can be designed to modulate various antimicrobial pathways [7]. Enhanced activity in both light and dark conditions can be accomplished by combining photocatalytically active materials with oxides that have inherent antimicrobial qualities [5]. Additionally, interactions between various oxides can enhance charge separation, inhibit electron-hole recombination, and encourage long-term ROS production, all of which can possibly contribute to increased antimicrobial efficacy [8].

Metal oxide particles for antimicrobial applications have been synthesized using a variety of techniques, such as solid-state processes, solvothermal and hydrothermal procedures, combustion synthesis, and co-precipitation [9]. Although these methods can yield materials with desired properties, they frequently have limitations such as difficulties of reproducibility, potential safety hazards, long synthesis times, etc. [10]. Because of their homogeneity and adjustable shape, powders synthesized by solution-based techniques are very useful for antibacterial applications. The sol–gel method is one of the approaches that allows for control over phase distribution, particle size, and composition, which makes it suitable to develop hybrid oxide systems [11,12].

It is well known that titanium dioxide materials possess antibacterial and antifungal activities [13,14,15]. Khashan et al. [16] have examined the antibacterial efficacy of TiO_2_ nanoparticles, synthesized by laser ablation, against a variety of Gram-positive and Gram-negative bacteria—*S. aureus*, *E. coli*, *P. aeruginosa*, and *P. vulgaris*. According to the authors, the size dispersion of the nanoparticles decreased as the laser ablation time increased. The concentration of 1000 µg mL^−1^ of TiO_2_ nanoparticles (NPs) had the strongest antibacterial effect against *E. coli*. Additionally, when coupled with amoxicillin, the NPs showed a synergistic impact that improved the suppression of *S. aureus* and *E. coli* growth.

The sol–gel method was used to prepare TiO_2_ and Cu-doped TiO_2_ NPs, which were then tested for antibacterial activity [17]. NPs’ antibacterial efficacy against both *E. coli* and *B. subtilis* was evaluated. Due to the relatively low Cu content in the composites, Cu doping did not considerably increase the antibacterial activity, despite the fact that both materials showed noteworthy antibacterial activity. The efficacy was roughly 90% against *B. subtilis* and 80% against *E. coli*, demonstrating the inherent potential of TiO_2_ nanoparticles [17].

Hajipour et al. [18] synthesized TiO_2_/CuO nanocomposites with varying CuO loading using a precipitation method and assessed their antibacterial efficacy against *S. aureus*. The research results demonstrated that the antibacterial activity of the TiO_2_/CuO heterojunctions was considerably improved by increasing the content of CuO. This was ascribed to the improved visible-light activity and the synergistic interaction between the metal oxides.

Through the production of ROS, membrane rupture, DNA damage, and enzyme inhibition, tellurium NPs are becoming potent antimicrobial agents that successfully kill bacteria and fungi. They are promising for a variety of applications since they can also inhibit biofilm formation, encourage wound healing, and improve the effectiveness of antibiotics. For their broad therapeutic usage, ongoing studies attempt to enhance their stability and safety [19].

It is known that materials containing copper oxide NPs have a wide range of potential uses in the fields of electronic devices, photoconductivity, gas sensors, etc. They can also be used to create different types of food packaging and antibacterial films [20,21]. It has been found that Cu^2+^ ions released from the NPs can damage DNA, cause mutations, promote the production of ROS, and are the main source of their antibacterial activity [22].

When metal nanomaterials are introduced into living systems, they interact with the cellular structures and metabolites. For their various practical applications, it is essential to evaluate their safety or, on the contrary, to evaluate their potential usage as materials with antimicrobial and/or cytotoxic effects [23,24,25].

The free-radical reactions and the formation of ROS constitute vital metabolic processes that are responsible for maintaining homeostasis, functional activity, and adaptation of the organism. These products are assessed in evaluations after treatment with pharmaceuticals, nanomaterials, and other biological effectors. The slight imbalance in their concentrations can easily provoke the processing of oxidative stress, inflammation, and diseases [26,27,28].

Chemiluminescence is an analytical technique that enables monitoring of the concentration and kinetics of free-radical and ROS generation with minimal sample volumes in reactions accompanied by light emission. This method allows dynamic observation of the free-radical reactions and determination of their prooxidant/antioxidant (inhibitory) effect, thereby indicating the potential harmful or beneficial impact of the tested substance or material on the living organism [29]. The usually very weak signal associated with these reactions can be greatly amplified by physical and chemical activators (probes), such as luminol, lucigenin, and others. The automatic digital registration in these assays enables the recording of the reaction kinetics and the evaluation of parameters such as rate constants, quantum yields, and other indicators of the interactions between the tested substance and ROS, which are generated in the tested chemical model reaction [29,30].

It should be noted that nanomaterials often exhibit markedly different properties and behavior compared to the same material at the macro-scale. Therefore, characterization and description of the properties of all newly synthesized nanomaterials are always needed [31,32].

In this study, newly synthesized TiO_2_/TeO_2_/CuO powders were investigated and subjected to thermal treatment at 400 °C and 600 °C. To the best of our knowledge, data on sol–gel–derived materials within the ternary TiO_2_/TeO_2_/CuO system remain scarce, which further motivated our work. The aim of the work was to study the effect of CuO on the structural and antimicrobial activity of sol–gel-derived TiO_2_/TeO_2_/CuO powders. This specific oxide combination was selected to integrate metal-ion–mediated toxicity with redox-driven bactericidal mechanisms into a single hybrid material, with the aim of achieving synergistic antimicrobial effects.

## 2. Results and Discussion

### 2.1. XRD and SEM-EDS Characterization of the Synthesized Powdered Sample

**Figure 1:**
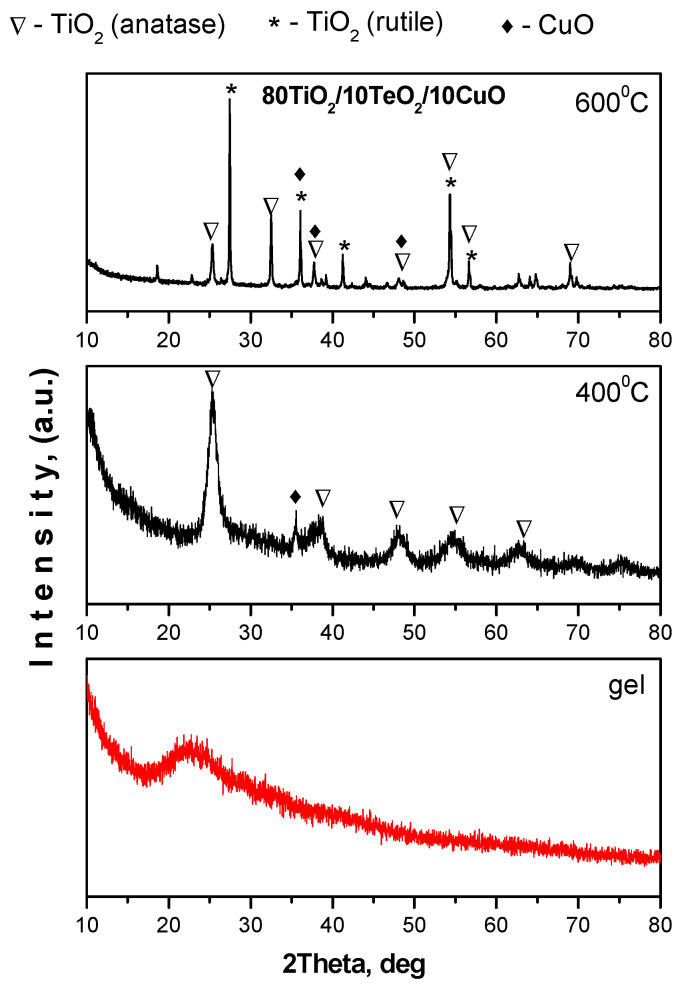
XRD patterns of the sample 80TiO_2_/10TeO_2_/10CuO before and after calcination at 400 °C and 600 °C.

The crystallinity and phase transformations of the analyzed ternary sample, which had been calcined at 400 and 600 °C, were investigated using XRD. The XRD patterns of the samples are shown in Figure 1. The graphic illustrates how the amorphous gel transforms into a crystalline after being heated to 400 and 600 °C for two hours of exposure time. As seen from the figure, at 400 °C, only TiO_2_ (anatase, JCPDS 78-2486) crystals have been found, and the highest peaks at 25.40°, 37.97°, and 48.20° were seen. At the higher temperature of 600 °C, multiple crystalline phases—anatase, rutile, and CuO—coexist within the sample. Among these, anatase remains the predominant phase. Due to the limited number of investigations on the ternary system, comparison of the obtained data was carried out mainly with studies on the corresponding binary TiO_2_-CuO system. XRD analysis of the TiO_2_–CuO binary system generally reveals the coexistence of crystalline TiO_2_ and CuO phases without the formation of new crystalline compounds in the composite structure [33,34].

Crystallite growth was evaluated by calculating the crystallite size of the TiO_2_ nanoparticles using the Scherrer equation of the sample heat-treated at 400 °C. The analysis indicates an average crystallite size in the range of 10–30 nm. According to the literature survey, XRD studies of the TiO_2_–CuO binary system have shown that TiO_2_ typically remains nanocrystalline, with crystallite sizes generally in the range of ~20–40 nm. Obviously, in this system, the presence of CuO has no significant effect on the TiO_2_ particle size [35,36]. On the other hand, it has been found that in the other binary TiO_2_–TeO_2_ system, the TiO_2_ crystallites generally exhibit larger dimensions (~60 nm) compared to those in TiO_2_–CuO systems, likely due to the influence of TeO_2_ on crystal growth during synthesis or heat treatment [37].

#### SEM Imaging and EDS Compositional Analysis

**Figure 2:**
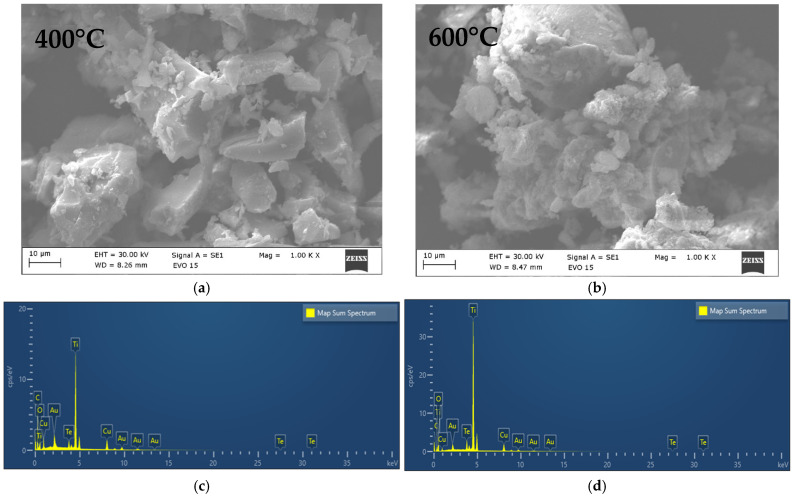
SEM/EDS images of the investigated sample heated at 400 °C (**a**,**c**) and 600 °C (**b**,**d**).

SEM imaging (Figure 2a,b) and EDS spectra (Figure 2c,d) were employed to verify the morphology and elemental distribution of the sol–gel–produced material. The SEM micrographs of the sample heat-treated at 400 °C (Figure 2a) reveal particles with well-defined morphologies alongside agglomerated grains exhibiting variations in shape and size exceeding 1 µm.

At elevated temperature (600 °C) it has been observed that higher particle agglomeration is characteristic of sol–gel–derived materials and is likely associated with their high surface reactivity. According to the EDS spectra (Figure 2c,d) of both heated powders, all elements—Ti, Te, Cu, and O—have been found in the powders. The absence of detectable impurities confirms the successful synthesis and high purity of the material.

**Figure 3:**
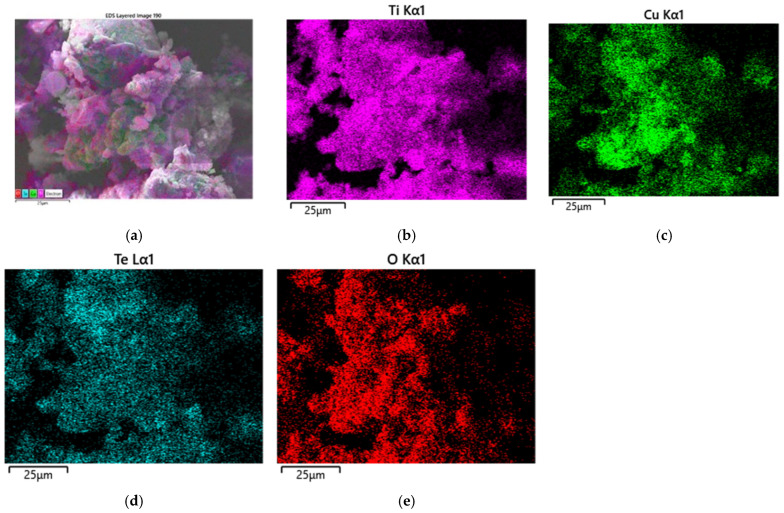
SEM image elemental mapping of the investigated sample heat treated at 600 °C (**a**); composition map of Ti (**b**); composition map of Cu (**c**); composition map of Te (**d**); composition map of O (**e**).

The corresponding energy-dispersive X-ray (EDS) elemental mapping (Figure 3a–e) confirmed the presence of the primary elements Ti, Te, Cu, and O in the sample heat-treated at 400 °C. A homogeneous spatial distribution of Ti, Te, and O was also observed, which has been preserved at 600 °C as well. Furthermore, the elemental maps indicate that Cu and Te were successfully incorporated into the TiO_2_ matrix during the sol–gel synthesis and the subsequent thermal treatment at 600 °C. Similar investigations in other systems containing TiO_2_ and CuO in the prepared composites demonstrate that SEM—EDS is effective in revealing particle morphology and elemental distribution in mixed oxide nanostructures [36,38].

Table 1 presents the semi-quantitative results of the chemical composition obtained by the EDS analysis. Generally, the measured composition is reasonably consistent with the nominal formulation, and the observed differences are within the typical uncertainty range of EDS measurements. The slight differences in the measured composition could be due to the low concentration of Cu and Te or to possible volatilization during processing.

### 2.2. UV–Visible Spectral Characterization

**Figure 4:**
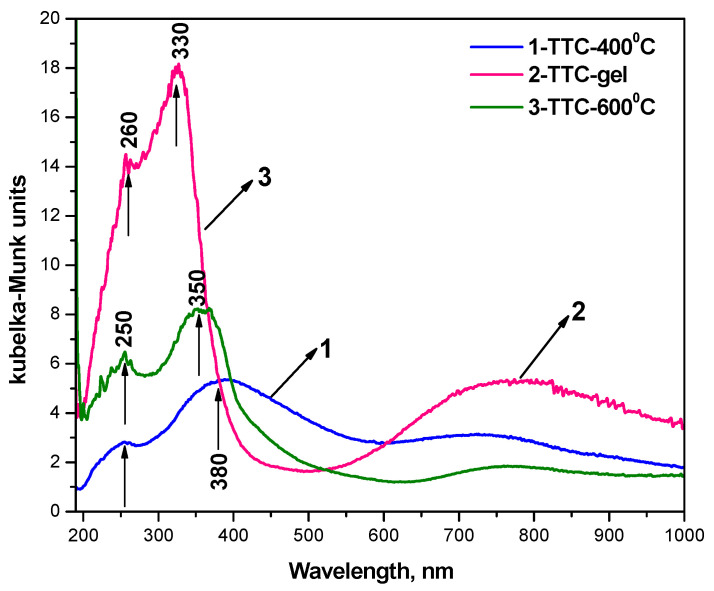
UV–Vis spectra of the investigated samples.

The optical absorption spectra of the investigated gel composition and heat-treated samples (at 400 and 600 °C) in the 200–1000 nm range are presented in Figure 4. As shown in the figure, the gel exhibited the highest absorption in the UV region, whereas the heated samples displayed a marked decrease in absorbance. Several characteristic bands were seen in all investigated samples: 250–260 nm, 330–350 nm, and a broad band in the range 600–900 nm. The bands at 250–260 nm are attributed to the ligand-to-metal charge-transfer transitions from O^2−^ to Ti^4+^ ions, associated with isolated or weakly connected TiO_4_ structural units [39,40]. The intensity of these bands decreases upon annealing at 400 °C and 600 °C, which could be connected to the progressive condensation and polymerization of the Ti–O and Te–O networks. The band centered near 330–350 nm could be assigned to the charge-transfer transitions involving TiO_6_ octahedra [41]. The progressive broadening of this band with increasing temperature suggests the conversion of tetrahedral Ti species into more condensed octahedral coordination environments [42,43].

A weak but broad absorption tail extending into the visible region (400–900 nm) is observed, particularly in the gel and heated at 400 °C samples. According to Choudhury et al. [44], the absorption between 400 and 500 nm appears as a result of interfacial charge transfer from the O 2p valence band to the Cu(II) state attached to TiO_2_. They also stated that these Cu(II) states may be present either as Cu(II) nanoclusters or in the form of an amorphous oxide phase of CuO. The absorption hump extending above 550 nm is mainly due to the d–d transition of Cu^2+^ in the crystalline environment of TiO_2_ [45,46]. On the other hand, such a broad absorption hump above 500 nm could be related to the C-modified TiO_2_ compositions [42,43]. It has also been reported that carbon could be another strong factor responsible for the visible-light absorption above 550 nm [47]. Obviously, the presence of copper as well as carbon shifts the absorption edge from the UV to the visible region.

The band gap (BG) of the investigated samples has been determined as well. Table 2 shows the band gap of the investigated samples compared to pure TiO_2_. From UV–vis spectroscopy, it could be seen that the temperature increase led to a reduction in the band gap. It is well known that in pure TiO_2_, the electronic transition occurs directly from the VB to the CB [33,34,37]. However, on Cu doping, the electrons are not directly excited to the CB since the unoccupied Cu^2+^ states and oxygen vacancies capture the electrons. This is the main reason for the reduction in the band gap of TiO_2_ particles.

On the other hand, it has also been stated that CuO presence is responsible for the red shifting of the absorption edge [48]. It could be summarized that the absorption edge shifting and band gap reduction are controlled by the surface of the nanoparticles, lattice strain, and vacancies [44].

### 2.3. Antimicrobial Property Results

The TiO_2_/TeO_2_/CuO hybrid powders’ antibacterial efficacy was tested against representatives of Gram-positive and Gram-negative bacteria—*Staphylococcus aureus* ATCC 25923 and *Escherichia coli* ATCC 25922, respectively. To guarantee a comprehensive assessment of the material capabilities, a combination of experimental strategies (spot assays and broth microdilution tests) was applied. The results discussed below reflect the bacterial growth after 24 h of incubation. The presented values are the average of three trials, and all MIC and MBC determinations were carried out in triplicate.

**Figure 5:**
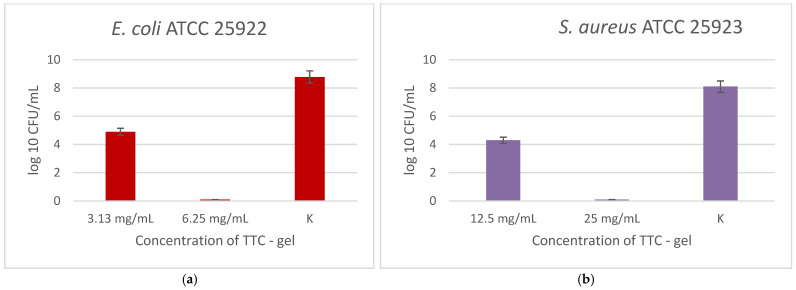
Antibacterial effect of TTC–gel, tested at different concentrations against *E. coli* (**a**) and *S. aureus* (**b**). Experiments were performed in triplicate as independent biological replicates.

At first, the antibacterial efficacy of TTC–gel was evaluated, as shown in Figure 5, against both bacteria. There was a noticeable concentration-dependent decrease in viable cells for *E. coli*. At 3.13 mg/mL, growth inhibition was observed compared to the control (K). The MBC was determined to be 6.25 mg/mL, where no viable cells were detected. A similar trend was observed for *S. aureus*. Bacterial viability significantly decreased at the lower tested concentration (12.5 mg/mL) in comparison to the K, while complete growth suppression was seen at 25 mg/mL, which corresponds to the MBC value of TTC–gel.

**Figure 6:**
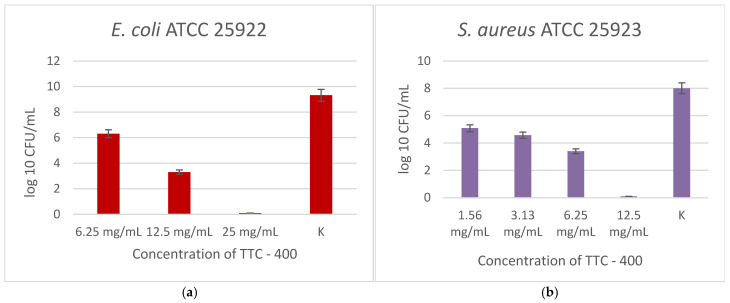
Antibacterial effect of TTC–400, tested at different concentrations against *E. coli* (**a**) and *S. aureus* (**b**). Experiments were performed in triplicate as independent biological replicates.

Figure 6 shows the antibacterial activity of the TTC sample calcined at 400 °C against *E. coli* (a) and *S. aureus* (b). Again, a concentration-dependent decrease in viable cells was noted for both bacteria. For *E. coli*, the MBC value was determined to be 25 mg/mL, as there was complete suppression of bacterial growth. Bacterial growth was significantly decreased at 6.25 and 12.5 mg/mL in comparison to the control.

There was a more noticeable antibacterial action against *S. aureus*. With increasing concentration, CFU/mL gradually decreased, and the MBC value was established to be 12.5 mg/mL. Inhibition was observed even at the lower concentrations compared to K. According to these findings, sample TTC–400 exhibited greater activity against the Gram-positive representative than the Gram-negative one.

**Figure 7:**
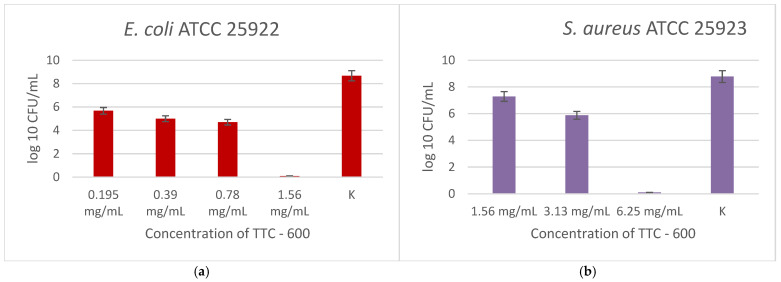
Antibacterial effect of TTC–600, tested at different concentrations against *E. coli* (**a**) and *S. aureus* (**b**). Experiments were performed in triplicate as independent biological replicates.

Figure 7 illustrates the antibacterial effect of the TTC sample calcined at 600 °C. For *E. coli*, a progressive suppression of bacterial growth was observed. At 1.56 mg/mL, complete bactericidal activity was attained, and no viable cells were discovered. Likewise, *S. aureus* showed susceptibility that was dependent on concentration, and complete inhibition was noted at 6.25 mg/mL, corresponding to the MBC value. However, compared to the TTC–400 sample, higher concentrations were needed to reach the bactericidal effect for the Gram-positive strain.

The sample calcined at 400 °C exhibited stronger antibacterial action against *S. aureus* and required a lower dosage for total eradication as compared to *E. coli*. Phase composition differences could be the cause of this contrast. According to the XRD analysis, while TTC–600 consisted of a mixture of anatase, rutile, and CuO phases, TTC–400 was primarily composed of the anatase phase. Pure anatase at 400 °C may be responsible for the Increase in surface reactivity, which could explain the higher antibacterial activity (Table 3).

### 2.4. Chemiluminescent Redox Activity Tests

**Figure 8:**
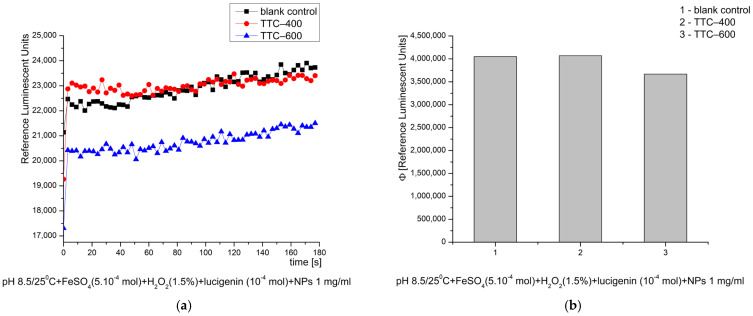
Effect of the synthesized nanomaterials on chemiluminescence in a system generating ·OH and ·OOH radicals at pH 8.5/25 °C, shown as kinetic curves (**a**) and as quantum yields (**b**).

In the Fenton’s system, at pH 8.5—optimal for the reaction—the newly synthesized nanomaterials exhibited no stimulation of the ROS generation over time. TTC–600 exhibited approximately 10% inhibitory activity towards ·OH and ·OOH radicals compared to the control reaction, an effect defined as antioxidant. TTC–400 showed no such effect (Figure 8). One possible explanation for the inhibitory activity is the adsorption and stabilization of generated radicals and ROS on the surface of the nanocomposite. The thermal treatment of the tested materials also influences their physical and chemical properties, which determined their behavior in the investigated reactions.

**Figure 9:**
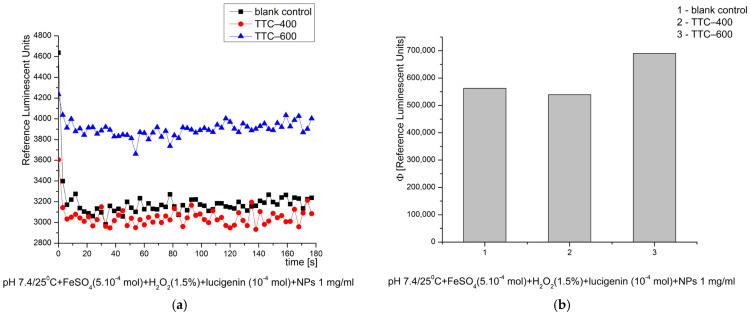
Effect of the synthesized nanomaterials on chemiluminescence in a system generating ·OH and ·OOH radicals at pH 7.4/25 °C, shown as kinetic curves (**a**) and as quantum yields (**b**).

In the Fenton’s system, at physiological pH 7.4, the exact opposite activity was observed: TTC–600 presented a pronounced prooxidant effect (~23%) when compared to the control reaction. TTC–400 showed neither pro- nor antioxidant activity (Figure 9). It had no effect on the generation of ·OH and ·OOH radicals with regard to the pH level. However, TTC–600 shifted its activity from slightly inhibitory to strongly prooxidant when pH was switched by approximately one unit (from 8.5 to 7.4) and dramatically altered the properties and activities of that nanomaterial in terms of ROS generation and sensitivity.

**Figure 10:**
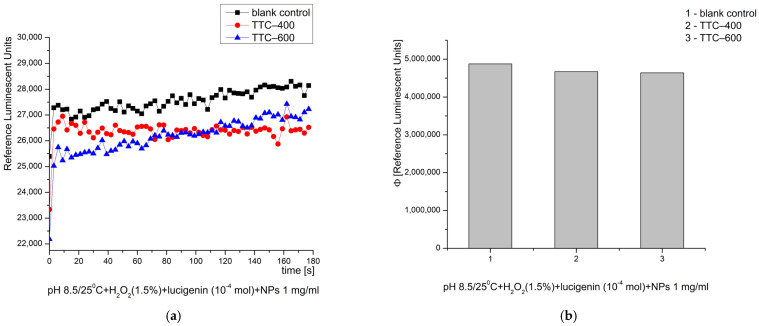
Effect of the synthesized nanomaterials on chemiluminescence induced by H_2_O_2_ at pH 8.5/25 °C, shown as kinetic curves (**a**) and as quantum yields (**b**).

In the system with hydrogen peroxide (H_2_O_2_) at pH 8.5, both tested nanomaterials exhibited a slight inhibitory effect, ~5%, compared to the control reaction (Figure 10). They stopped and even prevented the oxidation induced by that strong oxidant and ROS.

**Figure 11:**
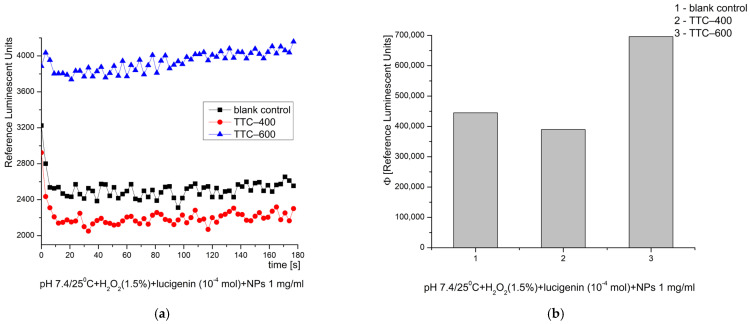
Effect of the synthesized nanomaterials on chemiluminescence induced by H_2_O_2_ at pH 7.4/25 °C, shown as kinetic curves (**a**) and as quantum yields (**b**).

The antioxidant activity was maintained and even enhanced at pH 7.4 (physiological) for TTC–400 (>12%). The other newly synthesized nanomaterial, TTC–600, exhibited pronounced prooxidant properties compared to the control reaction, ~56%, converting and strongly inducing its activity in the presence of hydrogen peroxide—a signaling molecule involved in the body’s immune response (Figure 11).

**Figure 12:**
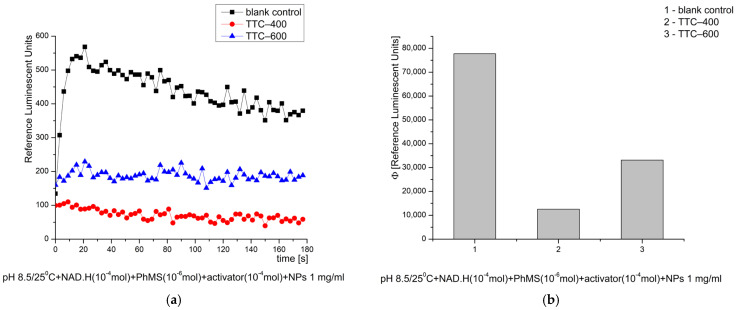
Effect of the synthesized nanomaterials on chemiluminescence in a system generating O_2_·^−^ radicals at pH 8.5/25 °C, shown as kinetic curves (**a**) and as quantum yields (**b**).

In the model chemical system for O_2_·^−^ radical generation, at pH 8.5, both tested nanomaterials exhibited strong inhibitory (antioxidant) activity as follows: TTC–400 ~84% and TTC–600 ~58%, when compared to the control blank reaction (Figure 12). That result suggests their potential application as “scavengers” of the most reactive and harmful free radical and ROS, which is always generated in significant concentrations when the cascade of free-radical oxidation starts and expands.

**Figure 13:**
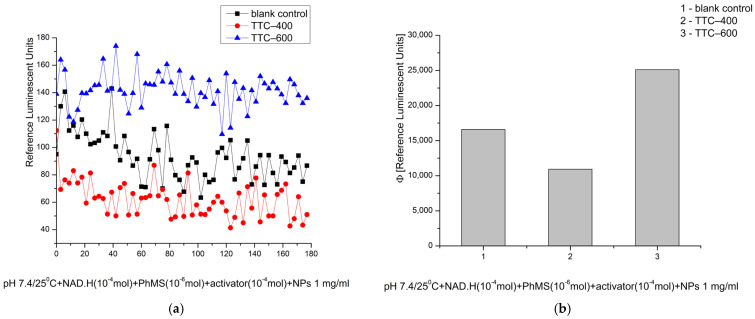
Effect of the synthesized nanomaterials on chemiluminescence in a system generating O_2_·^−^ radicals at pH 7.4/25 °C, shown as kinetic curves (**a**) and as quantum yields (**b**).

The observed effective inhibitory properties of TTC–400 were also confirmed at pH 7.4. The registered signal showed more than a 34% reduction when compared to the control blank reaction. However, TTC–600 exhibited >51% prooxidant activity when compared to the control reaction, totally converting the observed effect at pH 8.5 from inhibitory to oxidative at pH 7.4—physiological (Figure 13). It is important to note that in this model system, at both pH levels, the relative activities of the materials remained exactly the same, although their oxidizing properties compared to the control reaction changed.

In summary, at pH 8.5 (optimal), the as-prepared nanomaterials were most effective against superoxide radicals, neutralizing them extremely efficiently. At physiological pH 7.4, TTC–600 exhibited strong prooxidant properties, promoting the generation of all the various types of ROS to which its reactivity was tested—hydroxyl (·OH), hydroperoxyl (·OOH), and superoxide (O_2_·^−^) radicals—and also enhanced the oxidation with hydrogen peroxide (H_2_O_2_). That could be explained by both its involvement in redox-mediated reactions catalyzing the formation of secondary reactive species because of the changing valency metals in its content, as well as the changed chemical properties due to the higher thermal treatment.

The positive correlation between the increased level of ROS, respectively prooxidant activity, and enhanced antimicrobial efficacy is strongly supported by experimental and mechanistic studies. ROS, such as superoxide (O_2_·^−^) and hydroxyl (·OH) radicals and hydrogen peroxide (H_2_O_2_), induce oxidative stress that disrupts essential cellular structures. Materials with pronounced prooxidant behavior catalyze ROS production through surface redox cycling or Fenton-like reactions, thereby inducing bacterial oxidative damage [49,50].

The ROS-induced damage is non-specific. It is simultaneously targeting lipids, proteins, carbohydrates, and nucleic acids. Therefore, materials that significantly enhance ROS production and accumulation exhibit strong prooxidant properties. These translate into high antimicrobial activity through oxidative stress and irreversible cellular dysfunction [51].

It could be generalized that this study represents an initial attempt to clarify the possible mechanism underlying the antibacterial effects of the tested nanomaterials and their relationship to redox activity. The results indicate a clear correlation: materials exhibiting higher prooxidant activity tend to demonstrate enhanced antimicrobial efficiency. However, the mechanistic basis of this relationship remains insufficiently understood, and additional experiments are required to elucidate this observation. The obtained results imply that each material should be chosen for different applications with antibacterial or bacteria-preserving potential.

## 3. Conclusions

Nanopowders in the ternary TiO_2_/TeO_2_/CuO system have been successfully prepared by the sol–gel method. The XRD patterns showed that the presence of CuO delayed the anatase to rutile phase transformation at 600 °C. The SEM micrographs showed that particle agglomeration increased at 600 °C. EDS analysis confirmed the elemental compositions of the prepared powders. UV–Vis spectroscopy indicated that CuO is responsible for the red shifting of the absorption edge. Antibacterial tests revealed stronger *E. coli* inhibition with increasing calcination temperature and exposure time. The sample heat-treated at 600 °C exhibited the highest antimicrobial activity due to improved crystallinity and CuO dispersion for *E. coli*. All investigated samples demonstrated interesting antibacterial behavior. Sample TTC–400 demonstrated better antibacterial properties against the Gram-positive strain—*S. aureus*, having in mind the lower concentrations used as compared to the Gram-negative *E. coli*. Nevertheless, sample TTC–600 showed superior bactericidal properties against both bacterial strains. The observed antibacterial properties highlight the potential of the prepared powders for healthcare, sanitation, and antimicrobial surface coating applications. At physiological pH, TTC–600 exhibited strong prooxidant properties, which may contribute to its observed antimicrobial activity. At alkaline conditions, the newly synthesized nanomaterials were most effective against superoxide radicals. The variation in oxidative activity with changes in pH is indicative of the potential for controlled application of the synthesized materials, depending on the specific practical requirements and conditions.

## 4. Materials and Methods

### 4.1. Gel Preparation

A sample with the nominal composition 80TiO_2_/10TeO_2_/10CuO (mol%) was selected for detailed investigation. The gel was synthesized using Te(VI) acid (99.99%, Aldrich, St. Louis, MO, USA), Ti(IV) butoxide (≥99%, Fluka AG, Buchs, Switzerland), and copper sulfate pentahydrate (CuSO_4_.5H_2_O) (Merck, Darmstadt, Germany) as precursors, all dissolved in ethylene glycol (C_2_H_6_O_2_) (99%, Aldrich, St. Louis, MO, USA) with the molar ratio of 1:1. The well-documented issue of the rapid hydrolysis of Te(VI) alkoxides [52,53] was addressed by employing telluric acid (H_6_TeO_6_) instead of tellurium alkoxide. The precursor solutions were vigorously stirred for 5–10 min at room temperature to ensure complete dissolution. No water was intentionally added; ambient moisture served as the hydrolysis source for the sol–gel reaction. Depending on the composition, the pH values ranged from 4 to 5, and gelation occurred immediately for all investigated formulations. The gels were subsequently aged in air for several days to allow hydrolysis to reach completion. The dried gels were then calcined at 600 °C for two hours and cooled in air to obtain the final powdered material. The choice of calcination temperature was based on our previous findings [52]. The investigated samples have been denoted as TTC–400 and TTC–600.

### 4.2. Samples Characterization

Powder XRD patterns were collected at room temperature using a Bruker D8 Advance diffractometer (Berlin, Germany) equipped with Cu Kα radiation (λ = 1.54056 Å), a LynxEye position-sensitive detector, and an X-ray tube operated at 40 kV and 40 mA. Diffraction data were recorded over the 2θ range of 5.3–80° with a step size of 0.02°. The crystallite size of the sample was estimated from the XRD peak broadening using the Scherrer equation, which relates the crystallite size to the full width at half maximum (FWHM) of the diffraction peak. This approach is commonly used for estimating crystallite sizes in nanostructured materials [54].

Morphological observations were performed using a JSM-5510 scanning electron microscope (JEOL Ltd., Tokyo, Japan) operated at an accelerating voltage of 10 kV. Prior to imaging, the samples were coated with carbon using a JFC-1200 fine coater (JEOL USA Inc., Peabody, MA, USA). Elemental analysis by energy-dispersive X-ray spectroscopy (EDS) was conducted on a Zeiss Evo 15 microscope equipped with a Bruker detector (resolution 126 eV, Berlin, Germany).

Optical absorption spectra of the powdered samples were recorded in the 200–800 nm wavelength range using an Evolution 300 UV–Vis diffuse reflectance spectrophotometer (Thermo Electron Corporation, Madison, WI, USA), with magnesium oxide serving as the reflectance standard. The band gap energies (e.g., of the samples) were calculated by Planck’s equation: $E_{g}=\frac{h.c}{\lambda }=\frac{1240}{\lambda }$, where, e.g., is the band gap energy (eV), h is the Planck’s constant, c is the light velocity (m/s), and λ is the wavelength (nm) [55,56,57].

### 4.3. Antimicrobial Activity Testing

Two reference bacterial strains, *Escherichia coli* ATCC 25922 and *Staphylococcus aureus* ATCC 25923, were utilized to assess the samples’ antibacterial qualities. These strains are frequently linked to hospital-acquired infections and a variety of antibiotic resistance mechanisms. They are clinically significant examples of Gram-positive and Gram-negative pathogens, respectively.

Although both qualitative screening and quantitative antimicrobial activity determination were carried out during this experiment, the discussion only concentrates on the quantitative results.

The broth microdilution method was used to determine the minimum inhibitory concentration (MIC) and minimum bactericidal concentration (MBC). The criterion for bacterial suspensions was 0.5 McFarland, or roughly 1.5 × 10^8^ colony-forming units per milliliter (CFU/mL).

The culture medium used was Mueller–Hinton broth. To guarantee uniform suspension, the powders were distributed in the same media using an ultrasonic bath (Sonorex Bandelin, Berlin, Germany) running at 35 kHz for 15 min at room temperature. To achieve the desired concentration range, serial twofold dilutions were prepared.

The testing was performed in sterile 96-well microtiter plates, with each well containing both bacterial suspension and powder solution at the desired concentration. Included were sterility control wells and growth control wells—media only and bacterial suspension without powders, respectively. The plates were incubated aerobically for 18–24 h at 35 ± 2 °C. The presented values are the average of three trials, and all MIC and MBC determinations were carried out in triplicate.

Following incubation, the antibacterial activity was assessed by performing tenfold serial dilutions of samples from wells, followed by plating onto Mueller–Hinton agar. Subsequent to incubation, the following formula was used to determine the CFU/mL.

$$\mathrm{CFU}/\mathrm{mL}=\frac{(\mathrm{number}\;\mathrm{of}\;\mathrm{colonies}\;\times \;\mathrm{dilution}\;\mathrm{factor})\;}{\mathrm{volume}\;\mathrm{of}\;\mathrm{inoculum}},$$

When compared to the control (K), the MBC was determined as the lowest dose of powder that produced a 99.9% decrease in viable CFU/mL, demonstrating a bactericidal effect. The MIC, which denotes microbial inhibition, was established as the lowest concentration at which no visible growth was evident.

### 4.4. Activated Chemiluminescence Assay

The materials were applied at a final active concentration of 1 mg/mL in the reaction. Their effects on model free-radical oxidation reactions were quantified. All measured and target products represent endogenously formed metabolites, whose hyperconcentrations lead to oxidative stress and are generated during inflammation, immune response, or the progression of various diseases. The registered signal was compared to a control reaction in the absence of a nanomaterial (blank).

(1) The effect of the newly synthesized materials on the kinetics of free-radical oxidation reactions in buffered ex vivo model systems was tested at pH 7.4 (physiological) and at pH 8.5 (favoring radical generation), at 25 °C. The three reactions were monitored and recorded using the activated chemiluminescence method:
  - Fenton’s reagent (FeSO_4_-H_2_O_2_) for the generation of hydroxyl (·OH) and hydroperoxyl (·OOH) radicals;
  - Hydrogen peroxide (H_2_O_2_)—a strong oxidant and an ROS;
  - NAD.H–phenazine methosulfate system (PhMS) for the generation of superoxide radicals (O_2_·^−^).
(2) Statistical Analysis

All obtained data were analyzed using the independent *t*-test to identify the statistically significant effects. The observed effects were calculated as quantum yields, representing the integral value of the chemiluminescent response. The measured differences were considered significant at *p* ≤ 0.05. The data were registered by LUMIstar Omega (BMG Labtech GmbH, Ortenberg, Germany, 2020).

Methodology

System 1

The sample contained 0.2 M sodium hydrogen phosphate buffer adjusted to the chosen pH. The Fenton’s reagent consisted of FeSO_4_ (5 × 10^−4^ mol) and H_2_O_2_ (1.5%), and the chemiluminescent probe lucigenin (1 × 10^−4^ mol) was also added to the reaction mixture. Free radicals and ROS were generated according to the following interactions:

Fe^2+^ + H_2_O_2_ → Fe^3+^ + ·OH+ ^−^OH (1)

Fe^3+^ + H_2_O_2_ → Fe^2+^ + ·OOH + H^+^ (2)

System 2

The sample contained 0.2 mol sodium hydrogen phosphate buffer adjusted to the chosen pH, H_2_O_2_ (1.5%), and the chemiluminescent probe lucigenin (1 × 10^−4^ mol). Hydrogen peroxide served both as an oxidizing agent and an ROS.

System 3

The sample contained 0.2 mol sodium hydrogen phosphate buffer adjusted to the chosen pH, NAD.H (1 × 10^−4^ mol), phenazine methosulfate (1 × 10^−6^ mol), and the chemiluminescent probe lucigenin (1 × 10^−4^ mol). The scheme for the generation of superoxide radicals in this chemical model system is as follows:

PhMS + NAD.H + H^+^ → PhMS.H_2_ + NAD^+^

PhMS.H_2_ + PhMS → 2PhMS.H·

PhMS.H· + O_2_ → PhMS + O_2_^.−^ + H^+^

## Figures and Tables

**Table 1:** A comparison between nominal and measured (EDS) composition.

Nominal Composition, at %				Measured (EDS) Composition, at %			
Ti	Te	Cu	O	Ti	Te	Cu	O
27.59	3.45	3.45	65.52	17.83	1.27	4.41	76.49

**Table 2:** Investigated TiO_2_/TeO_2_/CuO samples, observed cut-off, and calculated optical band gap values (Eg).

Composition	Cut-Off, nm	Eg, eV
TiO_2_	387	3.2
TTC–gel	390	3.18
TTC–600 °C	410	3.02

**Table 3:** Minimum inhibitory concentration (MIC) and minimum bactericidal concentration (MBC) values of the investigated materials against the tested bacterial strains were determined by the broth microdilution method. Lower MIC and MBC values indicate higher antimicrobial activity.

Sample	*S. aureus* MIC	*S. aureus* MBC	*E. coli* MIC	*E. coli* MBC
TTC–gel	12.5 mg/mL	25 mg/mL	3.13 mg/mL	6.25 mg/mL
TTC–400	6.25 mg/mL	12.5 mg/mL	12.5 mg/mL	25 mg/mL
TTC–600	3.13 mg/mL	6.25 mg/mL	0.78 mg/mL	1.56 mg/mL

## Data Availability

The original contributions presented in this study are included in the article. Further inquiries can be directed to the corresponding author.
